# Can digital economy compensate the effect of aging on total factor productivity?

**DOI:** 10.1371/journal.pone.0301500

**Published:** 2024-04-18

**Authors:** Fange Meng, Xin Wen

**Affiliations:** 1 School of Economics, Capital University of Economics and Business, Beijing, China; 2 National Academy of Innovation Strategy, China Association for Science and Technology, Beijing, China; East China Normal University, CHINA

## Abstract

In China, the number of senior citizens has grown, along with the burden of old age, and aging has hampered economic growth. The advent of the digital age has led to the emergence of the digital economy as a new engine for economic growth. This paper uses DEA-Malmquist index model to measure the total factor productivity growth rate of 31 provinces in China from 2011 to 2021, and uses the moderating effects model to empirically investigate the relationship between the digital economy, aging and total factor productivity, and to verify whether the development of the digital economy can mitigate the negative impact of aging on total factor productivity. The results show that aging inhibits total factor productivity growth, and the digital economy can promote total factor productivity growth. Digital economy can alleviate the negative impact of aging on total factor productivity growth, and has a moderating effect. Digital economy plays a moderating role by improving the level of human capital and facilitating technological progress. The regional heterogeneity analysis shows that the moderating effect of the digital economy exists in the eastern and western regions and the southern region, but not in the central region and the northern region. Furthermore, the digital economy has a moderating effect on both the high and low aging groups. The research in this paper not only helps to evaluate the productivity effects of the digital economy, but also has important implications for finding ways to mitigate the negative effects of aging.

## Introduction

Population aging is a significant global issue [1]. The overall number of senior individuals 65 years of age and above is expected to surpass 700 million for the first time in 2020, reaching 723.484 million according to the World Bank’s Global Elderly Population Database. Among them, China has 168.864 million elderly people, which accounts for 23.34% of the global elderly population. It is currently the only nation on Earth with more than 100 million senior citizens. According to data from China’s National Bureau of Statistics, China has entered an aging society since 2000, the aging level reached 14.2% in 2021, entering the moderately aging society,which has only experienced 20 years. In contrast, in the United Kingdom, Switzerland, France, the process of aging to a moderately aging society took 47 years, 85 years, 115 years [2]. Compared with developed countries, China is aging rapidly. Moreover, when the phenomenon of aging appeared in developed countries, the level of per capita GDP had already exceeded 10,000 US dollars, while the level of per capita GDP when aging appeared in China in 2000 was less than 1,000 US dollars, showing the obvious characteristic of "aging before getting rich". There are obvious differences in the degree of population aging between regions in China, with the degree of aging in the eastern region significantly higher than that in the central and western regions, and the degree of aging in the southern region higher than that in the northern region. As the total fertility rate declines, fewer children are born, and life expectancy continues to rise, China’s future aging level will deepen. As the number of elderly people increases, the number of labor force begins to decline, bringing about a series of problems detrimental to economic growth, such as rising labor costs, increasing pressure on pensions, and slowing down the rate of human capital accumulation. Aging also reduces total factor productivity [3]. The age structure of China’s population has shifted. The demographic dividend is gradually disappearing. China’s economic growth rate is slowing down. In order to realize the second hundred-year goal, at this stage China needs to find a new point of economic growth to improve total factor productivity and promote economic growth. And the application of a series of information technologies, such as artificial intelligence and big data, promotes the development of digital economy. In the digital era, the digital economy can increase total factor productivity [4], bringing new opportunities for economic growth. The study of the relationship between aging, digital economy and total factor productivity in the context of the transformation of the age structure of the population is of great significance in promoting China’s economic growth and realizing the second hundred-year goal.

Digital globalization has brought new opportunities, and digital technology plays a key role in the process of globalization. The G20 Initiative on Development and Cooperation in the Digital Economy was adopted at the 2016 Hangzhou Summit. This initiative introduces the concept of the digital economy, which is defined as the use of digitalized knowledge and information as the primary factors of production and the use of contemporary information networks as a major carrier. It also refers to a range of economic activities where efficient use of ICT is a key factor in efficiency improvements and economic structure optimization [5]. The combination of modern digital technologies such as robotics, 5G, artificial intelligence, and ICT has driven the development of digital economy. The digital economy is integrating and permeating with the real economy through the application of new technologies such as big data, cloud computing, blockchain, and the Internet of Things [6]. The China Academy of Information and Communications Technology (CICT) released the Global Digital Economy White Paper, which contains data indicating that by 2022, the combined value of the digital economies of 51 countries will reach 41.4 trillion U.S. dollars, growing at a nominal annual rate of 7.4% and accounting for 46.1% of GDP. In terms of scale, the size of the U.S. and China’s digital economy is 17.2 trillion dollars and 7.5 trillion dollars respectively, ranking first and second in the world. Between 2011 and 2021, China’s digital economy expands at a quicker rate, from 9.5 trillion yuan to 45 trillion yuan in value. The digital economies of the United States, Germany, and the United Kingdom collectively accounted for over 65% of GDP. With growth rates of more than 20%, the digital economies of Saudi Arabia, Norway, and Russia are among the top three in the world. The digital economy is now having a significant influence on improving GDP, streamlining the economic structure, and bolstering global competitiveness.

China relied on the demographic dividend and technological progress to achieve rapid economic development [7]. Nowadays, as aging deepens and serious childlessness occurs, the absolute and relative number of China’s labor force begins to decline, the total dependency ratio rises, and the change of population age structure changes the advantage of population quantity dividend. The level of human capital has increased, and the quality of the labor force has improved, which can make up for the lack of labor force quantity supply. The construction of digital infrastructure promotes the development of the digital economy, and the development of information technology such as the Internet builds a platform for the dissemination of knowledge, which is conducive to knowledge spillover and enhances the level of human capital and technological progress. Human capital and technological progress are important factors affecting the growth of total factor productivity [8, 9]. The digital economy plays an increasingly important role in economic growth.

China’s economy is facing a "new normal". There is an urgent need to improve total factor productivity for economic growth against the backdrop of China’s aging population. Therefore, this paper focuses on the following three questions: first, does aging and digital economy affect total factor productivity growth? Second, if there is a negative impact of aging, can the digital economy have a moderating effect to mitigate the negative impact of aging? What is the path of the digital economy’s moderating effect? Third, is there a difference in the role of the digital economy in different regions? Clarifying the relationship between aging, digital economy and total factor productivity growth is of great significance for China as well as other developing countries to solve the aging problem and achieve economic growth. The contribution of this paper is mainly reflected in the following: first, in terms of research perspectives, most of the literature analyzes the impact of the digital economy on total factor productivity from the perspective of microenterprises, and few of them analyze the impact of the digital economy on regional total factor productivity at the macro level. Second, in terms of research ideas, this paper explores the negative impact of digital economy to alleviate aging and its functioning mechanism, which enriches the research literature on the relationship between digital economy, aging and total factor productivity, and provides ideas for solving the aging problem. Third, taking into account the disparities in regional economic development, it compares the contribution of the digital economy to mitigating the adverse effects of aging in the northern and southern regions. This comparison facilitates the formulation of tailored recommendations based on regional differences and the advancement of coordinated regional development.

## Literature review and theoretical analysis

### Literature review

Many countries in the world have entered the aging stage. Aging has become a global challenge [1] and has attracted attention from various countries. Maestas et al. [10], Aiyar et al. [11], Lee [3], and Park et al. [12] respectively studied the productivity impact of aging in the United States, Europe, the OECD, and Korea and found that aging reduces total factor productivity. But some scholars have also come to the opposite conclusion that aging can promote productivity growth [13]. Moreover, aging brings about labor shortage, the increase in the cost of labor for enterprises, which will force enterprises to use capital technology to replace labor and improve the level of technology [13], which in turn promotes the growth of total factor productivity. The research on the impact of aging on total factor productivity growth has not been clearly concluded. Compared with developed countries, China’s aging is characterized by a large scale, rapid growth rate, and aging before getting rich [14]. In addition, China’s declining fertility rate has further deepened the degree of aging and increased the burden of old age. If no measures are found to alleviate the aging problem, China will face more serious pressure on its social security system and face more severe aging challenges than other countries [15].

The rapid development of the digital economy has opened up new opportunities for economic growth and is a necessity to enhance international competitiveness [16]. Digital in the production process, on the one hand, as a production factor into the production function to promote economic growth. On the other hand, the application of digital can change the proportion of traditional factor input [17], reduce market distortion and improve allocation efficiency, so as to improve the productivity of traditional production factors. When data is used as a factor, it differs from traditional factors in the following four aspects [18]. First, the data factor does not have scarcity and follows Metcalfe’s law with increasing marginal returns. Second, it is not subject to the limitations of traditional factors in terms of geographic space and time, realizing virtual substitution of land and some low-skilled job labor to save costs. Third, it lacks competition, with an increase in customers resulting in a zero marginal cost. Fourth, it can be combined with traditional factors to promote factors in high productivity industries or regions, increasing marginal productivity.

The measurement of digital economy indicators is the basis for digital economy-related analysis and has attracted the attention of scholars, government agencies and international organizations. There are mainly three kinds of digital economy scale measurement. One is the construction of digital economy indexes. The OECD [19] constructed the ICT and digital economy statistical index system. The U.S. Information Technology and Innovation Foundation compiled the New Economy Index [20]. Ojanpera and Graham [21] constructed the Digital Knowledge Economy Index. Second, the value added of digital economy is measured. The U.S. Bureau of Economic Analysis (BEA) defines the scope of the digital economy and measures the size of the U.S. digital economy such as value added and total output using the supply-use table [22, 23]. In addition, the Australian Bureau of Statistics ABS [24], Statistics New Zealand [25], Statistics Canada [26], and a number of international consulting firms have all measured digital economy Value added has been measured [27]. Third, the construction of satellite accounts. The OECD set up a consulting group for GDP measurement under the digital economy, put forward the digital trade dimension framework and the basic framework of satellite accounts for the digital economy, and compiled a supply and use table [28]. Based on the measurement results of digital economy indicators, scholars analyzed the role of digital economy in eliminating regional income disparities [29], improving the efficiency of public administration [30], facilitating the transition from traditional to renewable energy [31], greening total factor productivity [32], and other applications.

Scholars have studied the relationship between the digital economy and total factor productivity. Rehamn and Nunziante [33] use GMM to verify the positive impact of the development of the digital economy on TFP in Europe. Niftiyev [34] analyzes the growth rate of the ICT sector and digitalization level of the countries along the Belt and Road, such as Armenia, Azerbaijan, and Georgia, and concludes that different levels of development of the digital economy lead to differences in labor productivity in manufacturing. Greenstein and McDevitt [35] and Jiménez et al. [36] analyze the impact of the Internet on the economic growth of the United States and Mexico. Hu et al. [37] argued that China’s digital economy can promote the improvement of total factor productivity. The mechanism of the digital economy on total factor productivity is based on the following aspects. The development of digital economy can reduce innovation risk, alleviate financing constraints, enhance technological innovation ability, and then promote total factor productivity [38]. The use of the Internet and other applications eliminates the information gap to a certain extent, increases labor employment options [39], promotes the flow of factors of production between sectors and industries, and improves the efficiency of resource allocation. The use of Internet technology enables the dissemination of information across time and space, generating technological spillovers, and the processing and integration of distributed information can multiply information, thus promoting human capital accumulation [40]. Digital transformation can also promote the employment of high-skilled labor by reducing the demand for low-end workers and increasing the demand for high-end talents [41]. The digital economy builds a communication platform between producers and consumers, improves product transactions and matching, increases entrepreneurial opportunities, promotes innovation, and releases the dividends of high-quality economic development [42]. Through digital technologies such as artificial intelligence and cloud computing, industrial digitization is realized, and the digital economy is cross-integrated among the three industries, promoting the optimization and upgrading of industrial structure [31, 43], and increasing productivity. The establishment of big data pilot zones can increase total factor productivity by improving pure technological progress [44].

China’s large and fast-growing elderly population has a heavy burden of old age, which has a negative impact on economic growth. Whether the digital economy can increase total factor productivity and alleviate the negative impact of aging needs to be further tested. Zhang and Li [45] analyzed the role of digital economy in mitigating the negative impact of aging, but did not specifically analyze the path of digital economy in mitigating the negative impact of aging.

Literature has been published using mixed regression [4], DID model [44], the bounds testing approach of cointegration and error correction modelling [46] and other. The DID model is able to test the effectiveness of digital economy development policies such as the establishment of big data pilot zones. However, it does not fully satisfy the condition of random sampling when grouping [47, 48], and cannot accurately measure the digital economy. This paper measures the digital economy indicators according to what the digital economy includes, and uses the moderating effect model to verify whether the digital economy can mitigate the negative impact of aging on total factor productivity, and to test the path mechanism by which the digital economy works.

### Theoretical analysis

Population aging can lead to a decline in the supply of labor [49], increase the total dependency ratio, increase the dependency burden, reduce savings, affect capital accumulation [50], and possibly affect capital accumulation, and may also affect research and development investment, which is not conducive to technological progress. The transfer of labor from low-productivity sectors to high-productivity sectors decreases and allocative efficiency decreases. Aging brings about the aging of the age structure of the labor force, and the increase in the number of older workers is not conducive to the adoption of new technologies and methods. The continued withdrawal of experienced older workers from the market has an impact on productivity. Population aging may have a negative impact on total factor productivity.

Hypothesis 1: Aging inhibits total factor productivity growth.

The digital economy has a scale effect that reduces the cost of knowledge transfer [51], promotes knowledge spillover, raises the level of human capital, improves the level of technology and promotes innovation. The carrier platform support effect can improve total factor productivity by building an information exchange platform, unclogging information exchange channels, reducing information asymmetry and transmission time lag, lowering the risk of innovation, and promoting technological progress. The use of digital technology by highly skilled human capital increases productivity [52–54]. Population aging brings about a decline in the number of labor supply, while the digital economy is conducive to knowledge spillover, promoting the accumulation of human capital and increasing the effective supply of labor. And the digital economy is integrated with traditional industries, enterprise capital technology to replace the labor force, from reducing labor demand. Therefore, the digital economy can reduce the negative impact of aging.

Hypothesis 2: By raising the level of human capital and technological progress, the digital economy can lessen the negative impacts of aging on the increase of total factor productivity.

There are differences in the level of human capital between regions, the eastern region has a higher level of economic development, which is conducive to attracting the inflow of highly skilled personnel, the level of human capital is better than that of the central and western regions, and the human capital externalities play a role in promoting the economic growth of the eastern region, which strengthens the enhancement of the level of human capital. Different economic environments between the north and south regions, the southern region near the sea, unique geographical location, water transport facilitation, reducing transportation costs, earlier participation in international trade activities to develop an export-oriented economy, a high degree of marketization. The high level of human capital and the degree of marketization provide favorable conditions for the development of the digital economy, and the digital economy plays a different role depending on the level of human capital and marketization between regions.

Hypothesis 3: The digital economy plays a different moderating role in different regions.

## Research design

### Econometric modeling

In order to explore the relationship between aging, digital economy and total factor productivity, the following econometric models are established. First, a baseline regression model was established to examine the effects of aging and digital economy on total factor productivity growth:

$$TFP_{it}=\alpha_{0}+\alpha_{1}old_{it}+\alpha_{2}digital_{it}+\alpha Z_{it}+\xi_{i}+\varepsilon_{it}$$ (1)

In Eq (1), *TFP*_*it*_ is the total factor productivity growth rate, *old*_*it*_ and *digital*_*it*_ denote aging and digital economy, respectively, and *Z*_*it*_ denotes a series of control variables including physical capital, healthy human capital, industrial structure, foreign investment, and infrastructure level. *ε*_*it*_ denotes a random perturbation term.

Second, A moderating effect model is established to test whether digital economy has an impact on the relationship between aging and total factor productivity:

$$TFP_{it}=\alpha_{0}+\alpha_{1}old_{it}+\alpha_{2}digital_{it}+\alpha_{3}old_{it}\times digital_{it}+\alpha Z_{it}+\xi_{i}+\varepsilon_{it}$$ (2)

In Eq (2), the cross-multiplication term of *old*_*it*_ and *digital*_*it*_ is used to test whether the digital economy has a moderating effect, if the regression coefficient is significantly non-zero, then it indicates that there is a moderating effect of the digital economy on the relationship between aging and total factor productivity, if *α*_3_>0, then the digital economy has a positive moderating effect, if *α*_3_<0, then the digital economy has a negative moderating effect.

Finally, to test the path of the digital economy to mitigate the negative impact of aging, whether the digital economy plays a role in mitigating the negative impact of aging by affecting human capital and technological progress. On the basis of the significant moderating effect model, the following econometric model is established:

$$M_{it}=\beta_{0}+\beta_{1}digital_{it}+\beta Z_{it}+\xi_{i}+\varepsilon_{it}$$ (3)

$$M_{it}=\gamma_{0}+\gamma_{1}old_{it}+\gamma_{2}digital_{it}+\gamma_{3}old_{it}\times digital_{it}+\gamma Z_{it}+\xi_{i}+\varepsilon_{it}$$ (4)

$$TFP_{it}=\theta_{0}+\theta_{1}old_{it}+\theta_{2}digital_{it}+\theta_{3}M_{it}+\theta_{4}old_{it}\times digital_{it}+\theta Z_{it}+\xi_{i}+\varepsilon_{it}$$ (5)

If *α*_3_ in Eq (2) is significant, it indicates that the digital economy can affect the relationship between aging and total factor productivity. In Eqs (3) (4) (5), M_it_ is the mediating variable, for human capital(*edu*_*it*_) and technological progress (rd_it_), if *γ*_3_ and *θ*_4_ are significant, it means that the digital economy plays a moderating effect by affecting human capital and technological progress, and then mitigates the negative impact of aging.

### Selection of variables

The explained variable is total factor productivity, and the DEA-Malmquis method was chosen to calculate the total factor productivity growth rate. DEA uses linear programming techniques to measure the performance of homogeneous decision-making units to maximize the amount of output and minimize the amount of input in the presence of fixed factors and technological environments, and is one of the methods that have been used to measure production efficiency [55, 56]. The Malmquist index method is based on the performance of each unit in the presence of two points in time the change in production preface of each unit at two points in time, and calculates the ratio of the rate of technical progress and the rate of change in technical efficiency [57]. The DEA-Malmquist method first builds a DEA model and then calculates the Malmquist index. The,Malmquist index for each unit is then compared and analyzed to derive the changes and trends in the performance of different units at different points in time. Gross regional product is the output variable, physical capital and the number of labor force employed are the input factors, and the method of calculating physical capital referred to the method of Zhang et al. [58]. Gross regional product and physical capital are converted to the base period level of 2000.

The core explanatory variable is aging, measured by the share of the population aged 65 and over in the total population.

The moderating variable is the digital economy. This paper refers to Huang et al. [59] to establish digital economy indicators. The digital economy is measured by two major indicators, namely, Internet development and digital financial inclusion, and the Internet development indicators include the number of Internet broadband access households (10,000), the number of urban units employed in the information transmission, software and information technology services industry (10,000), the total amount of telecommunications business (100 million yuan), and the number of end-of-year cell phone subscribers (10,000), while the digital financial inclusion indicators are measured by the Digital Financial Inclusion Index. The five indicators were analyzed synthetically using principal component analysis to finally obtain the digital economy measurement index.

The mediating variables are education human capital and technological progress. Educational human capital, measured by average years of schooling [60]. The levels of education are categorized into five levels, no schooling, elementary school, lower secondary school, upper secondary school, and tertiary and above, with years of schooling being 0, 6, 9, 12 and 16 years, respectively, multiplying the number of people in each level of education over the age of 6 years by the number of years of schooling at each level, and then summing up the values and dividing them by the total number of people over the age of 6 years to obtain the average years of schooling. Patent applications measure technological progress [61]. The number of patent applications can reflect the state of technological progress in a country or region.

#### Control variables

The following indicators were selected as control variables. Physical capital. The input and use of capital still has a large role in total factor productivity, and physical capital is derived using the calculation method of Zhang et al. [58]. Healthy human capital. As the level of development increases and the pace of life accelerates, health problems directly affect the time the labor force spends participating in the labor force, affecting productivity. Health human capital is measured using the number of beds in medical institutions. Industrial structure. The optimization and upgrading of industrial structure is an important reason for productivity, and industrial structure is measured using the share of total output value of the tertiary industry in regional GDP. Foreign Investment. Foreign investment can affect productivity by improving trade structure, introducing high technology, etc. Use the share of total investment of foreign-invested enterprises in regional gross output value. Infrastructure. Improvements in transport infrastructure can reduce transaction costs, facilitate the movement of factors between regions, optimize resource allocation and improve efficiency. The level of infrastructure is expressed in terms of the number of miles of road routes.

### Data description

The sample timeframe of this paper is chosen from 2011 to 2021, and panel data from 31 provinces and regions in China are used for empirical analysis. The data used come from the China Statistical Yearbook and provincial statistical yearbooks. The specific data are described in Table 1. Overall, the means and medians of the variables are close to each other, and the data have low dispersion and are evenly distributed, with no outliers that deviate from the overall picture.

**Table 1:** Descriptive statistics of variables.

variable name	variables	Obs	Mean	Std.Dev	Min	Median	Max
**total factor productivity**	tfp	341	0.9850	0.040	0.8754	0.9816	1.099
**digital economy**	digital	341	0.6000	0.067	0.486	0.589	0.886
**aging**	old	341	10.6952	2.774	4.824	10.33	18.8
**human capital**	edu	341	8.9289	1.089	4.222	8.885	12.54
**technology progress**	rd	341	10.4109	15.1213	0.017	4.92	98.0634
**physical capital**	terp	341	49.7036	8.887	32.66	49.08	83.73
**Health human capital**	pc	341	12.6033	6.307	2.917	11.32	42.37
**industrial structure**	hd	341	23.9869	15.893	0.84	20.83	72.13
**foreign investment**	fdir	341	0.6550	3.047	0.04762	0.2545	44.91
**level of infrastructure**	infr	341	0.9279	0.524	0.0514	0.9071	2.234

## Empirical analysis

### Benchmark regression

#### Impact of aging and the digital economy on total factor productivity

The baseline econometric regression model tests whether aging and the digital economy can affect total factor productivity growth. Considering the possible reverse causality between aging and total factor productivity, the econometric model is endogenous. The birth rate in the 1950s is used as an instrumental variable for aging. The population born in the 1950s is currently aging. The birth rate in the 1950s is not affected by total factor productivity during the sample study period, satisfying the instrumental variable selection criteria. The econometric model Eq (1) is estimated using 2SLS and the regression results are shown in Table 2.

**Table 2:** Benchmark regression results of aging and digital economy impacting total factor productivity.

	(1)	(2)	(3)	(4)
**digital**	0.1864**		0.3936***	0.7023**
	(2.0891)		(3.0735)	(2.0222)
**old**		-0.0069**	-0.0104**	-7.5987**
		(-2.0556)	(-2.3467)	(-2.2507)
**terp**	0.0001	0.0010	0.0003	0.5086
	(0.0719)	(1.3190)	(0.4410)	(0.4824)
**pc**	0.0012	0.0041***	0.0018*	0.3042
	(1.2698)	(4.2326)	(1.8278)	(0.2072)
**hd**	0.0006	0.0016***	0.0010*	-0.6969
	(1.1075)	(2.7198)	(1.7442)	(-0.8461)
**fdir**	0.0020***	0.0023***	0.0022***	0.8945
	(3.3855)	(3.8412)	(3.4817)	(1.0663)
**Infr**	-0.0239	-0.0056	-0.0218	-0.1070***
	(-1.0090)	(-0.2329)	(-0.8846)	(-3.1338)
**Constant**	0.8624***	0.9592***	0.8608***	0.1899**
	(23.1212)	(17.6784)	(13.4521)	(2.4142)
**N**	341	341	341	330
**R** ^**2**^	0.2787	0.4889	0.4642	0.1186

Note: 1%, 5%, and 10% significance levels are denoted by ***, **, and *, respectively; t-values are in parentheses; all regressions used fixed effects; same table below.

Column (1) in Table 2 shows the regression results of the digital economy on the total factor productivity. The regression coefficient of the digital economy on the growth rate of total factor productivity is positive and significant. The digital economy has a significant promotion effect on the growth rate of total factor productivity. This result is consistent with that of Pan et al. [4]. Column (2) in Table 2 shows that after considering endogeneity, the regression coefficient of aging on total factor productivity growth rate is significantly negative. Aging has a significant inhibitory effect on total factor productivity growth rate. Column(3) in Table 2 lists the results of both aging and digital economy. Aging has a significant negative impact on the growth rate of total factor productivity, while digital economy has a significant positive impact on the growth rate of total factor productivity. Hypothesis 1 is verified. Aging reduces the supply of labor, the transfer of surplus labor from the low-productivity agricultural sector to the high-productivity industrial sector decreases, and the flow of labor is impeded, which is not conducive to the improvement of the efficiency of resource allocation. The aging process reduces the supply of labor, reduces the transfer of surplus labor from the low-productivity agricultural sector to the high-productivity industrial sector, and impedes labor mobility, which is not conducive to the improvement of resource allocation efficiency. The development of the digital economy builds a platform for information circulation, breaking down the barriers to knowledge and information in time and space, which facilitates innovative activities and promotes technological innovation, thereby promoting growth in total factor productivity.

International organizations have different scopes of the digital economy. The measurement of digital economy indicators has not gained uniformity. This paper also draws on Bukht and Heeks [62], which divides the digital economy into the core, middle and outermost layers, and measures the digital economy using the entropy method from four perspectives: digital industry, digital innovation, digital users and digital activity. Due to the lack of data for Xizang, Xizang is not included when measuring the digital economy using the Bukht and Heeks method [62]. Column (4) in Table 3 shows the regression results of measuring the digital economy using the Bukht and Heeks method [62], and the digital economy is still able to significantly contribute to total factor productivity.

**Table 3:** Robustness test for the impact of aging and the digital economy on total factor productivity.

	(1)	(2)	(3)	(4)	(5)	(6)
	Substitution of variables			Delete area		
**digital**	0.1409*		0.3936***	0.1667*		0.4076***
	(1.7851)		(3.0735)	(1.7940)		(2.8286)
**old**		-0.0038*	-0.0104**		-0.0072**	-0.0107**
		(-1.8297)	(-2.3467)		(-2.1188)	(-2.2670)
**Constant**	0.9558***	0.9465***	0.8608***	0.8633***	0.9526***	0.8584***
	(28.9677)	(17.4680)	(13.4521)	(22.9820)	(17.2151)	(13.1435)
**Control variable**	YES	YES	YES	YES	YES	YES
**N**	341	341	341	330	330	330
**R** ^**2**^	0.1870	0.4974	0.4642	0.2749	0.4847	0.4574

Among the control variables, although the increase in the proportion of output value of the tertiary industry is favorable to total factor productivity growth, the result is not significant. China is in the stage of structural transformation, and economic growth has not yet completed the innovation transformation, and the promotion effect on the growth rate of total factor productivity has not been fully realized. Physical capital per capita has a significant positive impact on the total factor productivity. The knowledge spillover effect of the accumulation of physical capital formed by investment still plays an important role in the process of improving the total factor productivity growth rate. Healthy human capital has a significant positive impact on total factor productivity. Health level is related to the time of labor force participation in labor and the time of receiving education, and the improvement of health level helps to improve the labor participation rate and promote the accumulation of human capital, increase labor income, and improve the growth rate of total factor productivity. An increase in the proportion of foreign investment not only introduces advanced technology, but also promotes the exchange of highly skilled personnel between China and abroad, which is conducive to technological innovation and total factor productivity growth.

#### Robustness test of benchmark regression results

Substitute variables and deletion regions are used to test whether the results of Table 2 are robust. The results are shown in Table 3.

Columns (1), (2) and (3) in Table 3 use the method of replacing variables to test for robustness, with the level of aging replaced by the old-age dependency ratio for the share of the elderly population aged 65 and above, and the SFA method for calculating the growth rate of total factor productivity. Columns (4), (5) and (6) use the regression results with the data from Xizang deleted. Because in the selected timeframe, the proportion of the elderly population aged 65 and above in Xizang is below 7%, which does not meet the standard of an aging society. Xizang is deleted in the study of the relationship between aging and the growth rate of total factor productivity. The results in Table 3 show that under the two different test robustness methods of replacing variables and deleting regions, aging has a significant negative impact on total factor productivity growth rate, and digital economy has a significant positive impact on total factor productivity growth rate, and the results are similar to Table 2. The results in Table 3 show that aging has a negative effect on the total factor productivity growth rate and the digital economy has a positive effect on the total factor productivity growth rate, and the results are robust.

### Test of the moderating effect of the digital economy

#### An examination of the relationship between aging and total factor productivity influenced by digital economy

According to Eq (2), whether the digital economy plays a regulating role in the relationship between aging and total factor productivity is tested, and the results are shown in Table 4.

**Table 4:** Moderating effect test of digital economy.

	(1)	(2)	(3)	(4)
		Substitution of variables		Delete area
**digital**	0.0885	0.0893	0.1131	0.0045
	(0.7842)	(0.7874)	(1.3155)	(0.0391)
**old**	-0.0050	-0.0033	-0.0039	-0.0019
	(-1.1028)	(-1.0905)	(-0.8615)	(-0.5451)
**old×digital**	0.0937**	0.0963**	0.0852*	0.0730**
	(2.0908)	(2.0028)	(1.9017)	(2.0413)
**Constant**	0.9116***	0.8999***	0.9778***	0.9103***
	(13.1433)	(12.4381)	(7.3307)	(13.8804)
**Control variable**	YES	YES	YES	YES
**N**	341	341	330	330
**R** ^**2**^	0.3130	0.3039	0.1354	0.4015

Column (1) in Table 4 adds the cross-multiplier term between aging and the digital economy to the baseline regression to verify whether the digital economy affects the relationship between aging and total factor productivity. The results show that the coefficient of the cross-multiplier term of aging and total factor productivity growth rate is significantly positive at the 5% significance level. The digital economy can mitigate the negative impact of aging on total factor productivity. Aging brings a decline in the number of labor force and slows down the accumulation of human capital. The digital economy can strengthen information exchange, broaden data dissemination and sharing channels [63], and enhance the level of human capital. In addition, the digital economy can promote technological progress under the coupling effect [64], which leads to the replacement of labor by capital technology and enhancing total factor productivity and alleviating the negative impact of aging.

Use the method of replacing variables and deleting Xizang region to test whether the results of Column (1) in Table 4 are robust. The sample in column (2) in Table 4 is the replacement of the aging measure using the old age dependency ratio. Column (3) in Table 4 is the digital economy measured using the Bukht and Heeks methodology [62]. Column (4) in Table 4 deletes the Xinzang region. The regression results in columns (2), (3) and (4) in Table 4 show that the cross-multiplier term between aging and the digital economy is significantly positive. There is a moderating effect of the digital economy in the relationship between aging and total factor productivity, which can serve to mitigate the negative effects of aging. Therefore, the regression results in column (1) in Table 4 are robust.

#### Paths by which the development of the digital economy "hedges" against the suppression of total factor productivity growth by aging

According to Eqs (3), (4) and (5), the paper examines the path of the regulatory effect of digital economy and verifies whether digital economy directly regulates the relationship between aging and total factor productivity, or whether it regulates the relationship between aging and total factor productivity by influencing human capital and technological progress.

Columns (1), (2) and (3) in Table 5 test whether the digital economy plays a role in mitigating the negative effects of aging by affecting human capital. The regression results of the digital economy on human capital in column (1) in Table 5 show that the digital economy has a significant contribution to human capital. The digital economy facilitates the dissemination of knowledge and information, breaks down time-space barriers, and is conducive to improving human capital. Column (2) in Table 5 adds the cross-multiplier term of aging and digital economy on the basis of column (1), which is significantly positive. Column (3) in Table 5 adds the cross-multiplier term of aging and digital economy, and the cross-multiplier term is significantly positive. Columns (1), (2) and (3) in Table 5 indicate that the process of digital economy regulating aging and total factor productivity works through human capital.

**Table 5:** The path that the digital economy exerts the moderating effect.

	(1)	(2)	(3)	(4)	(5)	(6)
	edu	edu	tfp	rd	rd	tfp
**digital**	6.7170***	4.2036***	0.0599	4.7798***	9.2000	-0.1350
	(7.0250)	(3.8770)	(0.5386)	(5.6622)	(0.5508)	(-1.1599)
**old**		0.0152	-0.0044		-9.1908***	-0.0002
		(0.4271)	(-1.0407)		(-2.7116)	(-0.0813)
**old×digital**		1.0487***	0.0782*		12.7491***	0.0636**
		(3.1793)	(1.8984)		(2.6088)	(2.3163)
**M**			0.0074			0.0401***
			(1.2204)			(4.0200)
**Constant**	10.7238***	11.0725***	0.8315***	-2.1511***	102.3203***	1.0133***
	(17.2700)	(16.6261)	(8.7788)	(-3.9238)	(3.1837)	(16.1282)
**Control variable**	YES	YES	YES	YES	YES	YES
**N**	341	341	341	341	341	341
**R** ^**2**^	0.9248	0.9159	0.3725	0.9688	0.8834	0.4205

Columns (4), (5) and (6) in Table 5 test whether the digital economy plays a role in mitigating the negative effects of aging by influencing technological progress. The digital economy significantly contributes to technological advancement, according to the regression results of the digital economy on technological advancement in column (4) in Table 5. The digital economy can lower information gathering costs [51], encourage knowledge spillovers, and facilitate the dissemination of technology in support of technological growth. Column (5) in Table 5 adds the cross-multiplier term of aging and digital economy to column (4), which is significantly positive. Column (6) in Table 5 contains the cross-multiplier terms of aging and digital economy, which are significantly positive. The results in columns (4), (5) and (6) of Table 5 indicate that the process of the digital economy moderating aging and total factor productivity works through technological progress. The results in Table 5 verify the validity of Hypothesis 2.

The robustness of the results in Table 5 is tested using the replacement variable method. Drawing on Acemoglu [65] and Autor and Dorn [66], the human capital skill structure is measured using the ratio of the number of years of education of the labor force with a bachelor’s degree or higher to the number of years of education of the labor force with a college degree or lower. Technological progress is measured using R&D intensity. The results are presented in Table 6. The results in Table 6 are consistent with the results in Table 5, suggesting that the digital economy plays a role in mitigating the negative effects of aging by influencing human capital and technological progress.

**Table 6:** Robustness test for the path of the moderating effect.

	(1)	(2)	(3)	(4)	(5)	(6)
	edu	edu	tfp	rd	rd	tfp
**digital**	0.8306***	0.6084***	0.0283	2.0460***	1.1700***	0.0968
	(8.3526)	(6.6080)	(0.2900)	(4.7674)	(2.6977)	(0.9882)
**old**		0.0030	-0.0012		-0.1043	-0.0001
		(1.2074)	(-0.4483)		(-1.1856)	(-0.0516)
**old×digital**		0.0773***	0.0416*		0.2100*	0.0378*
		(3.7976)	(1.9283)		(1.6553)	(1.7400)
**M**			0.0510***			0.0115
			(3.8982)			(0.6493)
**Constant**	-0.0832	-0.0484	1.1201***	1.9265***	3.4354***	0.9198***
	(-1.2878)	(-0.8565)	(14.6710)	(6.9121)	(4.1171)	(15.4259)
**Control variable**	YES	YES	YES	YES	YES	YES
**N**	341	341	341	341	341	341
**R** ^**2**^	0.9587	0.9689	0.4442	0.9850	0.9866	0.4241

### A test of regional heterogeneity in the moderating effects exerted by the digital economy

Aging, digital economy and total factor productivity growth rate have different degrees of development in different regions, and there may be regional heterogeneity in the regulating effect of digital economy. In order to verify whether the regulating effect of digital economy is different in different regions, the 31 provinces are divided into eastern, central and western regions according to the level of economic development, and the 31 provinces are divided into southern and northern regions according to different geographic locations. According to the different levels of aging development, the sample is divided into a low-aging group and a high-aging group. The results of the regional heterogeneity regression are shown in Table 7.

**Table 7:** Test for regional heterogeneity in the moderating effects of the digital economy.

	(1)	(2)	(3)	(4)	(5)	(6)	(7)
	the east	the central	the western	the southern	the northern	low	high
**digital**	-0.2869*	-0.2184	0.4798**	-0.1538	0.1860	0.9171***	-1.1108***
	(-1.7966)	(-0.9413)	(2.4727)	(-0.9689)	(1.2738)	(4.1098)	(-3.6740)
**old**	-0.0004	0.0128**	-0.0094	-0.0205**	0.0036	-0.0153*	0.0023
	(-0.0996)	(2.5412)	(-1.3695)	(-2.0428)	(1.0868)	(-1.6794)	(0.5207)
**old×digital**	0.0909**	-0.0842	0.1245*	0.1251*	0.0283	0.5341***	0.2925**
	(2.1953)	(-0.9782)	(1.9237)	(1.9263)	(0.7556)	(2.9782)	(2.1097)
**Constant**	0.5349***	0.9142***	0.7327***	1.0307***	0.8077***	0.9444***	1.1075***
	(4.0514)	(10.5354)	(9.0080)	(12.1743)	(14.4115)	(7.6109)	(8.8575)
**Control variable**	YES	YES	YES	YES	YES	YES	YES
**N**	121	88	132	198	143	183	158
**R** ^**2**^	0.6563	0.5040	0.5545	0.1284	0.6176	0.3163	0.1851

Columns (1), (2) and (3) in Table 7 show the regression results of the test of the moderating effect of the digital economy in the eastern, central and western regions, respectively. The regression results show that the cross-multiplier terms of aging and digital economy are positive and significant in the eastern and western regions, while the cross-multiplier term in the central region is negative and insignificant. The digital economy plays a moderating effect in the eastern and western regions to mitigate the negative impact of aging on total factor productivity. The rapid economic development of the eastern region and the relatively perfect infrastructure are conducive to attracting talents, which provides favorable institutional and environmental conditions for the digital economy to play a role, and the digital economy has a significant moderating effect in the eastern region. The western region has a low degree of aging, and the development of the digital economy has brought more employment opportunities for the labor force in the western region, while the "East Counts, West Counts" project connects the western region with the eastern region, promotes the flow of digital elements, and brings new development opportunities for the western region, which is conducive to the high-quality development of the economy. While the economic development of the central region mainly relies on capital and labor, the pace of digital economic development has not been regulated without the corresponding talent and infrastructure. Hypothesis 3 is valid.

Columns (4) and (5) in Table 7 show the regression results of the test of the moderating effect of the digital economy in the southern and northern regions, respectively. The regression results show that the cross-multiplier term between aging and digital economy in the southern region is positive and significant, and the digital economy plays a moderating effect in the southern region. The cross-multiplier term between aging and digital economy in the northern region is not significant and there is no moderating effect. Although the total factor productivity growth rate and the level of digital economy development in the southern region and the northern region are closer. The aging degree in the southern region is slightly higher than that in the northern region. The economic and institutional environment and other factors in the northern region limit the moderating effect of the digital economy. The southern and northern regions have different degrees of openness to the outside world. The proportion of foreign investment in the southern region is much higher than that in the northern region. The northern region is deeply influenced by the planned economy, relying on factor investment to drive economic growth and develop heavy industry, with insufficient impetus for market-oriented reforms. The southern region relies on shipping and inland waterways to develop an externally oriented economy, and participates in the international market earlier, with a higher degree of marketization than that of the northern region. The level of transportation infrastructure in the North is lower than that in the South, with the mileage of highway routes in the North being only 0.64 km/km^2^, while in the South it is 1.34 km/km^2^, nearly twice that of the Northern region. The development of the service sector in the North is slow, and the industrial structure is unreasonable. Compared with the southern region, the northern institutional mechanism reform is lagging behind, the legal system and scientific awareness and other backward. The southern region provides a relatively relaxed development environment for the development of the digital economy, while the above series of problems limit the development of the digital economy in the northern region, which is not conducive to the digital economy to play the role of regulating the negative impact of aging.

Columns (6) and (7) in Table 7 show the regression results of the moderating effect test of the digital economy for the low and high aging groups. The results show that the cross-multiplier terms of aging and digital economy are positive and significant in both the low-aging group and the high-aging group. Regardless of the level of aging, the digital economy can play a role in mitigating the negative effects of aging. This result further verifies that the digital economy can alleviate the negative impact of aging on total factor productivity.

## Conclusion

During the period of deepening aging and economic structural transformation, it is of great practical significance to study the relationship between population aging, digital economy and total factor productivity to achieve China’s second hundred-year goal. The results of empirical analysis show that aging has an inhibitory effect on total factor productivity, while digital economy can significantly promote total factor productivity growth. The digital economy can affect the relationship between aging and total factor productivity and play a positive moderating role. The digital economy can improve human capital, promote technological progress, and slow down the negative impact of aging on total factor productivity. In terms of regional heterogeneity, the digital economy in the eastern and western regions has a moderating effect, while the digital economy in the central region has yet to show a moderating effect. The digital economy in the southern region has a moderating effect, while the digital economy in the northern region does not have a moderating effect. Moreover, the digital economy can play a moderating role regardless of the level of aging.

Based on the empirical findings, the following insights can be obtained. First, strengthening the construction of digital infrastructure, promoting the integration of the digital economy with the real economy, and realizing industrial digitization and digital industrialization. The construction of digital infrastructure is the foundation for the integration of the digital economy with the real economy. It further promotes the synergistic construction of 5G networks and gigabit optical networks, accelerates the development of the Internet of Things, and provides network infrastructure for digital applications. The government has improved relevant laws and regulations to provide a favorable environment for enterprises to participate in the construction of the digital economy. Second, regional differences should be fully taken into account, and different digital economy development strategies should be formulated according to the level of regional economic development, and achieve balanced regional development. In terms of regional heterogeneity, the digital economy in the eastern region has begun to play a moderating role and can alleviate the negative impact of aging. Therefore, the eastern region should further improve the level of investment in human capital, and enhance the ability of independent innovation, so as to provide tecnological support for digital and accelerate the level of development of the digital economy. The western region should make use of the "East Counts West Counts" project to improve economic growth. At present, the "East Counts West Counts" project faces problems such as a single big data industry structure and a lack of talent support. Talent links between the eastern and western regions should be strengthened to further explore cooperative projects and expand the industrial structure. The central region, where the digital economy has yet to play a regulating role, should focus its economic development on promoting economic growth, strengthening digital infrastructure construction, providing a foundation for the development of the digital economy, and providing favorable conditions for the digital economy to play its role. From the perspective of the North and South regions, the Northern region should improve the degree of marketization, increase R&D expenditures, strengthen the construction of digital infrastructure, and improve institutional mechanisms to provide a favorable economic environment for the development of the digital economy, so that the digital economy can fully play its role in improving the level of productivity and economic growth in the Northern region, and narrowing the gap between the economic growth of the Northern and Southern regions. Third, increase investment in human capital and upgrade the level of human capital. Talent is the core competitiveness of the country and is also related to the effect of the digital economy on promoting total factor productivity growth. The government should raise the level of human capital by extending the number of years of free education and increasing the number of laborers with higher education. China’s fertility rate is declining, and the number of educated people is decreasing accordingly. Therefore, this stage is a favorable opportunity to increase investment in education and extend the number of years of free education. Moreover, the study’s findings offer empirical evidence for countries experiencing severe aging to raise their digital economy levels.

## Discussion

This paper verifies that the digital economy can enhance human capital, promote technological progress, and mitigate the negative impact of aging on total factor productivity. However, there are some problems in the development of digital economy, such as digital freedom, digital divide, digital risk and so on. In the future, the elderly population will increase, while knowledge is updated quickly, how to help the elderly cross the digital divide is an important issue that needs to be paid attention to.
